# Effects of a co-designed exercise and sport intervention on cardiorespiratory fitness and metabolic syndrome components among individuals living in a refugee camp in Greece: A randomized controlled trial

**DOI:** 10.1016/j.jmh.2024.100227

**Published:** 2024-03-27

**Authors:** Florian Knappe, Konstantinia Filippou, Antonis Hatzigeorgiadis, Ioannis D. Morres, Emmanouil Tzormpatzakis, Elsa Havas, Harald Seelig, Sebastian Ludyga, Flora Colledge, Marianne Meier, Yannis Theodorakis, Roland von Känel, Uwe Pühse, Markus Gerber

**Affiliations:** a Department of Sport, Exercise and Health, University of Basel, Grosse Alle 6, Basel CH-4052, Switzerland; b Department of Physical Education and Sport Sciences, University of Thessaly, Trikala 42100, Greece; c Department of Nutrition and Dietetics, University of Thessaly, Trikala 42132, Greece; d Department of Health Sciences and Medicine, University of Lucerne, Lucerne 6005, Switzerland; e Interdisciplinary Center for Gender Studies, University of Bern, Bern 3012, Switzerland; f Department of Consultation-Liaison Psychiatry and Psychosomatic Medicine, University Hospital Zurich, University of Zurich, Zurich 8091, Switzerland

**Keywords:** Cardiovascular disease, Migrant, Physical activity, Public health

## Abstract

**Background:**

The metabolic syndrome epidemic, including in forcibly displaced individuals, requires cost-effective prevention and treatment strategies. Yet, the health needs of forcibly displaced individuals often remain underserved. Our study evaluated the effect of a co-designed exercise and sport intervention on cardiorespiratory fitness and metabolic syndrome components among individuals in a refugee camp in Greece and examined the indirect effect through cardiorespiratory fitness on metabolic syndrome components.

**Methods:**

We conducted a randomized controlled trial involving an intervention and a wait-list control group with *n* = 142 (52.8 % women) forcibly displaced Southwest Asians and Sub-Saharan Africans. The intervention group participated for 10 weeks in exercise and sport activities. Outcomes were cardiorespiratory fitness and single metabolic syndrome components. Effects were analyzed with structural equation modeling.

**Results:**

In total, 62.7 % of participants presented with low cardiorespiratory fitness levels (<40th percentile), and 24.6 % met the criteria for metabolic syndrome. In the intervention group, 73.5 % attended the exercise and sport sessions at least once a week. There was evidence for a direct intervention effect on cardiorespiratory fitness, ß_direct_ = 0.12, *p* = 0.022, but not for any of the metabolic syndrome components (*p* ≥ 0.192). Cardiorespiratory fitness significantly facilitated the intervention's indirect effect on abdominal obesity, ß_indirect_ = −0.03, *p* = 0.012, high diastolic blood pressure, ß_indirect_ = −0.04, *p* = 0.011, and elevated triglycerides, ß_indirect_ = −0.03, *p* = 0.025.

**Conclusion:**

Implementing exercise and sport activities in a refugee camp in Greece effectively reaches a wider target population and improves cardiorespiratory fitness among forcibly displaced individuals. The intervention contributes to a decrease in abdominal obesity, high diastolic blood pressure and elevated triglycerides indirectly via improved cardiorespiratory fitness.

## 1 Introduction

In 2018, ``The Global Epidemic of the Metabolic Syndrome'' (Saklayen, 2018) outlined the escalating public health challenge of cardiometabolic diseases. Core of the article was an estimated worldwide metabolic syndrome (MetS) prevalence of 25 %. This syndrome, characterized by a constellation of interrelated cardiometabolic risk factors, represents a substantial health concern (Honarvar et al., 2023). Data from the Global Burden of Disease study (Chew et al., 2023) illustrates the consequences, with obesity being responsible for 5.0 million deaths in 2019, hyperlipidemia for 4.3 million, type 2 diabetes mellitus for 1.4 million, and hypertension for 1.1 million. These conditions collectively led to 346.7 million disability-adjusted life years.

The prevalence of MetS is increasing globally (Saklayen, 2018) and recent reports (Al-Rousan et al., 2022; Rosenthal et al., 2022; World Health Organization, 2022) also emphasize an elevated risk among forcibly displaced populations compared to the host population. These findings contradict the reported ``healthy immigrant effect'' (Newbold, 2006), which states that only the most resilient individuals undertake the strenuous migration. Such an effect may not apply to forcibly displaced individuals who, especially in armed conflicts, represent a cross-section of the general population. However, forcibly displaced individuals constitute a diverse group in which the prevalence of major chronic diseases differs considerably between subgroups and host countries (Nisar et al., 2023).

Given the UNHCR's alarming report (United Nations High Commissioner for Refugees, 2023) of a doubling of forced displacement in the last decade, leading to 108.4 million affected people, the enhanced risk of cardiometabolic diseases in certain subgroups is of particular concern. While most individuals are internally displaced or find refuge in neighboring countries, forced migration to Europe tripled, resulting from human-made conflicts and natural disasters. Greece is a primary entry point due to its geographical proximity to Asia.

Considering the human right to health (World Health Organization, 1946) and the various benefits that healthy forcibly displaced individuals bring to host nations (World Health Organization, 2022), governments are called upon to prevent the emergence of health inequalities between host- and forced displaced populations. However, public health strategies are reported to overlook the health of forcibly displaced individuals (Matlin et al., 2018).

Due to the outlined magnitude, such strategies should be effective and affordable. Exercise and sport consistently demonstrate beneficial effects on various health outcomes, including cardiometabolic diseases (Pedersen and Saltin, 2015). Specifically, exercise and sport significantly improve MetS components and cardiorespiratory fitness (CRF), reducing cardiovascular risk (Pattyn et al., 2013; Batacan et al., 2017; Yang et al., 2009). Given this empirical evidence, international clinical guidelines emphasize the need for lifestyle interventions, including exercise, in primary prevention programs for cardiovascular diseases (Aerts et al., 2021). However, similar to the broader adult population (Mitchell et al., 2013), low exercise adherence among forcibly displaced populations (Elshahat and Newbold, 2021) could impact the effectiveness of these interventions.

Moreover, no evidence exists regarding the potential benefits of exercise and sport on MetS components among forcibly displaced individuals in refugee camps. Given the distinct contextual challenges, such as lived experiences and post-migration living difficulties, the reach and effectiveness of an exercise and sport intervention may be compromised.

This study evaluates the effect of a co-designed (Masterson et al., 2022) exercise and sport intervention on CRF and MetS components among individuals living in a refugee camp in Greece. Specifically, we aim to examine the direct intervention effects of exercise and sport on CRF and MetS components and the indirect intervention effect on MetS components through CRF among forcibly displaced individuals. Additionally, we seek to explore the extent to which individuals in a refugee camp in Greece utilize exercise and sport activities. We expect that an exercise and sport intervention will improve CRF and MetS components, with improvements in MetS components being attributed to the enhancements in CRF.

## 2 Method

### 2.1 Study design

This study was part of a larger pragmatic randomized controlled trial, including an intervention group and a wait-list control group. The study assessed the impact of an exercise and sport intervention on the physical and mental health of individuals living in a refugee camp in Greece (ISRCTN16291983). The present paper provides insight into the immediate post-intervention effects on CRF and MetS components following a 10-week intervention period. The sampling and procedure of this study aligned with those outlined in the registered project (Gerber et al., 2021). Ethical approval was granted by the Research Ethics Committee of the University of Thessaly, ref. approval no. 39.

### 2.2 Setting

The research was conducted in a refugee camp in Greece's central region. The camp was run under the governance of the Ministry of Migration and Asylum and served as a temporary residence for those awaiting the decision of their asylum applications. Individuals lived in container units, referred to as "households" in this study. Each household had cooking and sanitary facilities. For health concerns, a medical center, staffed with two doctors, nursing personnel, and two psychologists, delivered primary healthcare services. The camp was located in a rural area and was a 15–20 min walk away from the nearest village, which housed a small grocery store. A city could be reached within 25 min by bus, which ran three times a day. Adults were not legally allowed to engage in paid work.

According to the site management, the camp recorded 1376 individuals in February 2021. Of these, 920 (67 %) residents were aged between 16 and 59 years, and 39 % were women. The camp hosted a diverse mix of individuals regarding their socio-demographic backgrounds. A large proportion came from Afghanistan (45 %) and Syria (25 %). The remaining 30 % originated from West Asia (11 %), Sub-Saharan Africa (17 %), or other areas (2 %).

### 2.3 Participants

Those eligible to participate in the study included individuals who (a) lived in the selected refugee camp, (b) were between 16 and 59 years old, (c) were able to read or communicate in English, Arabic, Farsi, or French, and (d) provided written informed consent. For ethical reasons, and in accordance with the pragmatic trial, broad inclusion criteria were defined to enable as many individuals as possible to participate in the exercise and sport activities. Arabic, Farsi, and French represented most camp resident's linguistic backgrounds. A minimum sample size of 136 participants, with an expected dropout rate of 25 %, was estimated to detect an intervention effect on PTSD symptoms, the primary outcome of the parent study (Gerber et al., 2021). Since the power analysis accounted for a rather small effect size (*ƒ* = 0.15), the calculated sample size was considered sufficient for the present study, expecting a moderate effect (*ƒ* = 0.25) for CRF (Batacan et al., 2017; Milanović et al., 2015).

### 2.4 Procedure

We started the study in May 2021 with screening, recruitment, baseline assessment (T1), and random allocation. To prevent exclusion or separation of individuals within the same household, recruitment and allocation were performed by households. A broad overview of the sociodemographic profile of the camp population was obtained at the outset of the study by screening as many households as possible. A sample stratified by sex was then drawn from the pool of screened and eligible households. We drew extra households in case of non-attendance to ensure that we would reach the targeted sample size. Following T1, households were randomly allocated by the Greek project coordinator into two groups with a 1:1 allocation rate between groups and sexes. Accordingly, family households were randomly assigned to either the intervention or the wait-list control group using a random number generator. This process was then repeated, first for households consisting solely of female residents and finally for those with only male residents. Three days prior to the start of the intervention, study participants were informed about their group allocation by the coaches. Since participants of the control group were assigned to a waiting list, allocation concealment was not possible. Different project members carried out the screening, sampling, and allocation process, respectively, in order to mitigate selection bias.

Prior to data collection, we asked potential study participants to provide written informed consent. Participants received written and verbal information detailing the study's purpose and procedure. They were assured that their participation was voluntary and that withdrawal from the study would bear no negative consequences, especially concerning their asylum application process. This approach aimed to safeguard participants from potential harm, coercion, and exploitation. However, cultural and language differences, misunderstandings, and false expectations might have jeopardized voluntary participation (Spaaij et al., 2019). We therefore recruited 10 female and male community translators from the camp population to minimize this risk. They had to represent the study participant's origin and be proficient in English and one of the other study languages. Community translators received training prior to their involvement covering translation (e.g., verbatim translation, maintaining confidentiality) and the project's purpose. They took a crucial role in engaging with camp residents due to their familiarity with the camp resident's cultural backgrounds and the camp environment's specific context. The responsibilities of community translators included explaining study procedures, obtaining informed consent, assisting with translation, and supporting overall data assessment.

T1 and post-assessment (T2) took place at the Department of Physical Education and Sport Science of the University of Thessaly. We chose this location due to the requisite infrastructure and equipment availability. A shuttle bus transferred the participants to and from the testing site. After the assessment, participants were briefed about their results and referred to a specialist in cases where a health risk was identified. Furthermore, participants were compensated with a meal and sports equipment (worth about 40 euros). These measures contributed to the research having reciprocal benefits for participants.

### 2.5 Intervention

The intervention was implemented in a community setting. The standard of care consisted of the regular living conditions in the refugee camp. This included support from a social worker or a general practitioner on demand and participation in activities of low physical intensity (e.g., crafting classes or table soccer tournaments) organized by the site management support.

Aerobic exercise interventions of 12 or more weeks showed bigger improvements in CRF compared to shorter interventions (Batacan et al., 2017). The project's initial start was delayed due to COVID-19-related health risks and restriction measures. As a result, the intervention period had to be shortened from 12 to 10 weeks to carry out the assessment within the approved timeframe. Due to the potential positive impact, the exercise and sport intervention was continued and opened to the wait-list control group and all camp residents after the official trial ended.

The intervention was co-designed to support participant's agency and consider, adapt, and continuously optimize the intervention to individual, cultural, and situation-specific conditions. This approach emphasized the significance of user experience in developing and refining interventions (Masterson et al., 2022). Tailored interventions have further been increasingly recognized as promising approaches to enhance exercise participation (Ma et al., 2021). We defined co-design as a structured process that involves service users, staff, and camp management at every stage of quality improvement (Masterson et al., 2022). Co-design was implemented before and throughout the intervention, guiding the initial design and any subsequent modifications. We used qualitative screening tools such as free-listing and held numerous informal personal and group conversations with participants and stakeholders before the start of the intervention. Participant observation and feedback loops provided insights during the intervention trial to identify challenges early and take appropriate countermeasures. Aspects of the design and modifications primarily included the type of activity, content in the individual sessions, personal and group-specific goals, place and time of implementation, adaptation to changing climatic and structural conditions or measures to address barriers for participation (e.g., organizing a childcare service during the activities or providing a vision-protected room for women's activities).

Activities were offered 5 times per week for 50 to 60 min separately for women and men, including ball sports, resistance training, martial arts, and dancing. Participants were free to participate in their preferred activities and motivated to engage in them for a minimum of 2 days per week. Participants were regularly informed about upcoming activities and motivated to join through an internal chat group and direct, personal interactions. In accordance with the principles of self-determination theory (Ryan and Deci, 2000), participants were encouraged to engage in the activities at their own pace. Each session was provided by 2 postgraduate students in physical education of the same sex as the participants in collaboration with community translators. The coaches familiarized themselves with UNHCR's ``sport for protection'' toolkit (United Nations High Commissioner for Refugees, 2018) and received additional training in practically implementing the self-determination theory. The activities were implemented inside and near the camp in the cooler evening hours before Maghrib. After an agreement with the site management, it was possible to use existing structures such as a soccer field, volleyball court, gymnastics room, and an empty storage hall.

### 2.6 Measures

Trained research personnel executed the data collection of outcome variables, namely CRF and cardiovascular risk markers at T1 (week 0) and T2 (week 11). Data assessment was carried out following a standard operating procedure. Due to the nature of the intervention and the involvement of some assessors in the implementation of activities, blinding to group assignment was unfeasible. To ensure accurate understanding and instruction delivery, translators proficient in English, Arabic, Farsi, and French were present during data collection.

#### 2.6.1 Cardiorespiratory fitness

We employed the Åstrand–Rhyming Indirect Test of Maximal Oxygen Uptake to measure CRF using a bicycle ergometer (Åstrand and Rodahl, 2003). Peak VO_2_max (ml/kg/min) was calculated based on sex, an age correction factor, body weight, mean steady state, and power output (Buono et al., 1989). The Åstrand–Rhyming test's validity in estimating VO_2_max has been reported previously (Macsween, 2001), and the protocol has been employed with forcibly displaced individuals (Sundquist et al., 2010). Cut-offs adjusted for sex and age were used to differentiate between low (<40th percentile) and higher CRF (American College of Sports Medicine 2010).

#### 2.6.2 Metabolic syndrome components

To determine MetS components, we assessed cardiovascular risk markers. Waist circumference was measured using a measuring tape. A digital blood pressure monitor (Omron, Kyoto, Japan) was used to measure systolic and diastolic blood pressure three times within a 5-min interval, after a 5-min resting period in a seated position. The average of these 3 measurements was then computed. The validity of this device has been documented in prior research (Qi et al., 2021). A single finger prick was performed to collect approximately 10 blood drops for all (capillary) blood analyses. Blood samples were examined with an Afinion 2 analyzer (Abbott, Wädenswil, Switzerland) to assess fasting plasma triglycerides, high-density lipoprotein cholesterol (HDL-C), and the average blood glucose level over the previous 3 months (glycosylated HbA1c). The Abbott 2 point-of-care analyzer results have been shown to correlate well with reference laboratory tests for lipid levels and HbA1c (Abbai et al., 2017; Foerster and Severn, 2016).

MetS and its components were defined according to the joint consensus from several key organizations (Alberti et al., 2009). Criteria and cut-off points included abdominal obesity (waist circumference ≥80 cm for females or ≥94 cm for males), high blood pressure (>130 mmHg systolic BP or >85 mmHg diastolic BP), elevated triglycerides (>1.7 mmol/dL), lowered HDL-C (<1.3 mmol/L for females or <1.0 mmol/L for males), and elevated fasting glucose (>100 mg/dL). Instead of elevated fasting glucose, elevated HbA1c (>5.7 %) was used (Cavero-Redondo et al., 2019). MetS was diagnosed when 3 or more of these conditions were met.

#### 2.6.3 Adherence

The coaches consistently tracked the participation rate throughout the intervention period using attendance records. Attendance was recorded at every session separately for each participant and summarized as the total number of activities engaged in throughout the study. Adherence was evaluated based on frequency of attendance and was categorized as two or more, one, or less than one weekly participation.

#### 2.6.4 Sociodemographic background

A questionnaire was used to collect sociodemographic information from participants, covering sex, age, country of origin, educational background (none, primary, high school and above), and duration of stay in the camp (in months).

### 2.7 Statistical analysis

We conducted statistical analyses using SPSS (Version 29.0, IBM, Armonk, United States) and the AMOS interface for descriptive and inferential analysis. The data analysts were not blinded to the allocation of participants. Data were screened for uni- and multivariate outliers, applying the Mahalanobis distance criterion (Barnett and Lewis, 1994) for the latter. After visual inspection, Kolmogorov-Smirnov tests were performed to check Gaussian distribution. We applied log-transformation to variables that exhibited non-normal distribution and had skewness/kurtosis z-values exceeding 3.29 (Kim, 2013).

Sample characteristics and outcome measures are displayed as mean (M), confidence interval (95 % CI), frequency (n), and relative proportion (%). Data are presented separately for women and men, following UNHCR's recommendations in their latest sport strategy (United Nations High Commissioner for Refugees, 2022) and recent evidence indicating that women experience greater benefits than men from identical exercise doses in reducing cardiovascular mortality risk (Ji et al., 2024). We used Chi-square and independent *t*-tests to explore whether participants who completed T2 and women differed from dropouts and men regarding sociodemographic background or one of the outcome variables at T1. We analyzed further missing values with the Little's Missing Completely at Random Test (Little, 1988). Missing values of participants who completed both assessments were then substituted by the expectation-maximization method.

Anticipated dropout, stemming from the contextual circumstances of the camp setting where individuals might have needed to leave or chosen to depart, thus becoming unable to continue their participation in the treatment, led us to conduct a completer analysis. This approach was chosen assuming that the loss to follow-up occurred randomly and was not associated with either the treatment or the condition under study (Bell et al., 2013). By including nonadherent participants, we aimed further to reduce post-randomization confounding and selection bias in our findings.

**Fig. 1:**
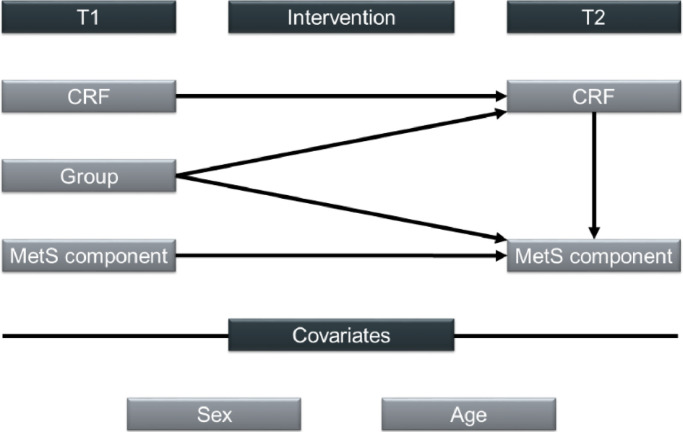
Model examining the direct intervention effects on CRF and MetS component and the indirect intervention effect through CRF on MetS component. Outcomes were controlled for sex and age.

*Abbreviations*: CRF, cardiorespiratory fitness; MetS, metabolic syndrome; T1, baseline; T2, post-intervention.

We used a causal model (Fig. 1) to evaluate the direct intervention effect on CRF and MetS components through path analysis controlling for T1 values. Sex and age were included as covariates in the analysis due to their association with MetS components (Hiramatsu et al., 2023). Additionally, we aimed to understand the pathways through which the intervention exerted its effects. We employed structural equation modeling to investigate multiple relationships simultaneously within a single model. The intervention's effects on MetS components were modeled through a direct and an indirect path via CRF. Unlike regression models, where variables are either dependent or independent, structural equation modeling allows variables to have both roles in different model components. Therefore, it facilitates the assessment of indirect effects in longitudinal data in a single analysis. Compared to regular regression analysis, structural equation modeling subsequently reflects causal pathways more accurately, including an expected direct and concurrent indirect effect and CRF's dual role as intervention outcome and facilitator of the intervention indirect effect (Gunzler et al., 2013). Direct and indirect effects of all variables were estimated using bias-corrected bootstrap confidence intervals derived from 10,000 bootstrap samples (Efron and Tibshirani, 1994). The null hypothesis that regression coefficients equal zero was tested and rejected at *p* < 0.05.

The model fit was examined using both absolute and relative fit indices. According to guidelines (Iacobucci, 2010; Browne and Cudeck, 1992), an acceptable fit was indicated by *χ*^2^/*df* < 3.00, CFI > 0.95, and RMSEA < 0.08. RMSEA values between 0.08 and 0.10 constituted a marginal fit.

## 3 Results

### 3.1 Sample

**Fig. 2:**
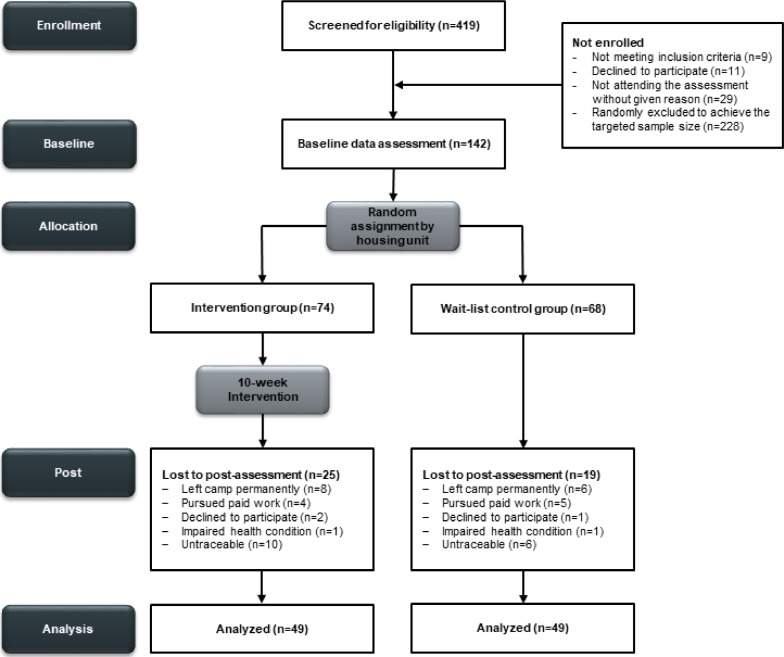
CONSORT flow diagram of the study.

Participant flow of the study is displayed in Fig. 2. The targeted 1:1 ratio was not wholly achieved due to the selection by household and stratification by sex. Out of 142 participants (75 women) at T1, 98 participants (57 women) completed T2 (dropout: *n* = 44, 31.0 %). The reasons for dropout were often unknown as the individual could not be traced anymore (*n* = 16, 11.3 %) or they left the camp permanently due to completion of their asylum procedure (*n* = 14, 9.9 %). Dropouts were more likely from West Asia, Central, or East Africa than South Asia, *χ*^2^(3) = 15.65, *p = 0.*001, and had higher HDL-C levels, *t*(51.4) = 2.06, *p = 0.*044, than participants who completed T2.

The sociodemographic background of the participants is presented in Table 1. Two-thirds (*n* = 89, 62.7 %) had low CRF (<40th percentile) according to the ACSM guidelines (American College of Sports Medicine, 2010) and a quarter (*n* = 35, 24.6 %) met the inclusion criteria for MetS. When considering age and sex-adjusted cut-offs for those who completed T2, women had statistically lower CRF, *χ*^2^(1) = 4.11, *p = 0.*043. Specifically, 27 (67.5 %) women were represented in the lowest CRF category (<20th percentile) compared to their male counterparts (*n* = 17, 44.7 %) at T1. Additionally, while women had a higher prevalence of abdominal obesity, *χ*^2^(1) = 25.82, *p* < 0.001, they exhibited reduced risks of high systolic, *χ*^2^(1) = 9.06, *p = 0.*003, and diastolic blood pressure, *χ*^2^(1) = 6.57, *p = 0.*010. Furthermore, they were less often affected by elevated triglycerides than men, *χ*^2^(1)=3.91, *p = 0.*048, at T1.

**Table 1:** Sociodemographic background and differences by group allocation.

	Overall (*n* = 142)	IG completers (*n* = 49)	WLCG completers (*n* = 49)
*Sex*			
Women	75 (52.8)	29 (59.2)	28 (57.1)
Men	67 (47.2)	20 (40.8)	21 (42.9)
*Age* (in years)	29.2 [27.7;30.8]	27.8 [25.1;30.5]	31.9 [29.0;34.9]
*Origin*			
South Asia	77 (57.0)	36 (75.0)	29 (60.4)
West Asia	18 (13.3)	6 (12.5)	4 (8.3)
East Africa	22 (16.4)	4 (8.3)	7 (14.6)
Central Africa	18 (13.3)	2 (4.2)	8 (16.7)
*Education*			
None	36 (26.5)	11 (22.4)	13 (27.6)
Primary	51 (37.5)	24 (49.0)	17 (36.2)
High school and above	49 (36.0)	14 (28.6)	17 (36.2)
*Time in camp (in months)*	14.9 [13.2;16.7]	13.9 [11.3;16.6]	15.5 [11.9;19.1]

*Note*: Data are presented as mean [95 % CI] and frequency (%).

*Abbreviations*: IG, intervention group; WLCG, wait-list control group.

After checking for outliers, pairwise values for waist circumference (*n* = 4), systolic blood pressure (*n* = 2), triglycerides (*n* = 2), and HbA1c (*n* = 8) with Mahalanobis distances of ≥14.2 and 1 implausible value of 91.9 ml/kg/min for CRF (>3 SDs above the mean) were removed. There were between 1 and 9 (1.0–9.2 %) missing values for the individual cardiovascular risk factors at T1 and between 2 and 6 (2.0–6.1 %) at T2, mainly due to measurement errors of the devices. In total, 20 (20.4 %) uni- and multivariate missing values for CRF were observed at T1 and T2, respectively, primarily due to acute injuries, knee complaints, or feelings of discomfort. Little's Missing Completely at Random Test was not significant, *χ*^2^(207) = 217.00, *p = 0.*303, indicating that the probability of missing data was the same in all cases.

### 3.2 Intervention

The activities could be carried out largely as planned despite the need for continuous adjustments to the changing contextual conditions (e.g., the camp management's alternate use of the training room or rising temperatures). No adverse events were reported. The average weekly participation rate was 2.0 (SD = 1.6). Overall, 20 participants (40.8 %) adhered to the recommended 2 weekly training sessions, and 16 individuals (32.7 %) participated once per week. There were no statistical differences in the participation rate, *t*(31.6) = −1.13, *p = 0.*266, between women and men.

### 3.3 Intervention effects

Table 2 presents the descriptive statistics for the outcome measures at T1 and T2. The direct and indirect effects on CRF and MetS components are displayed in Table 3 as standardized path coefficients. We observed a significant direct intervention effect on CRF at T2, ß_direct_ = 0.12, *p = 0.*022, 95 % CI: 0.02, 0.23. The intervention group increased their CRF at T2 by a mean of 2.8 ml/kg/min more compared to the wait-list control group. There was no evidence for a direct intervention effect on any of the MetS components (*p ≥ 0.*192). However, we found statistically significant indirect intervention effects through CRF on abdominal obesity, ß_indirect_ = −0.03, *p = 0.*012, 95 % CI: −0.07 to −0.00, high diastolic blood pressure, ß_indirect_ = −0.04, *p = 0.*011, 95 % CI: −0.08 to −0.01, and elevated triglycerides, ß_indirect_ = −0.03, *p = 0.*025, 95 % CI: −0.08 to −0.00, at T2. The model fit was acceptable overall, except for RMSEA, indicating a marginal fit for abdominal obesity, high systolic and diastolic blood pressure, and low HDL-C.

**Table 2:** Outcome measures at T1 and T2 for control and intervention groups, separated by sex.

	T1 (all)			T2 (all)		
	*n*	Control (*n* = 49)	Intervention (*n* = 49)	*n*	Control (*n* = 49)	Intervention (*n* = 49)
*CRF* (in ml/kg/min)	78	32.6 [29.2;36.1]	32.1 [28.6;35.7]	78	32.0 [28.6;35.4]	37.0 [33.3;40.7]
*Waist circumference* (in cm)	94	88.9 [84.2;93.6]	86.9 [82.4;91.3]	95	90.8 [87.0;94.6]	86.0 [81.7;90.3]
*Systolic blood pressure* (in mmHg)	96	120.4 [116.3;124.5]	118.2 [114.7;121.7]	95	118.4 [114.4;122.4]	117.7 [114.1;121.2]
*Diastolic blood pressure* (in mmHg)	97	82.5 [80.0;84.9]	80.1 [77.7;82.5]	96	80.1 [77.5;82.8]	78.1 [75.9;80.3]
*Triglycerides* (in mmol/L)	89	1.7 [1.4;1.9]	1.6 [1.4;1.8]	93	2.2 [1.8;2.5]	2.0 [1.7;2.3]
*HDL-C* (in mmol/L)	90	1.2 [1.1;1.3]	1.1 [1.1;1.2]	94	1.1 [1.0;1.2]	1.0 [1.0;1.1]
*HbA1c* (in %)	89	5.4 [5.3;5.5]	5.4 [5.3;5.5]	92	5.4 [5.3;5.5]	5.3 [5.2;5.4]

	T1 (women)			T2 (women)		
	*n*	Control (*n* = 28)	Intervention (*n* = 29)	*n*	Control (*n* = 28)	Intervention (*n* = 29)
*CRF* (in ml/kg/min)	40	27.2 [21.7;32.7]	26.4 [22.4;30.4]	40	25.48 [21.5;29.5]	33.7 [28.2;39.3]
*Waist circumference* (in cm)	53	91.9 [85.6;98.2]	88.5 [82.2;94.8]	54	92.1 [86.7;97.5]	86.5 [80.2;92.9]
*Systolic blood pressure* (in mmHg)	55	116.0 [110.5;121.5]	112.1 [107.9;116.4]	54	116.1 [110.4;121.7]	112.4 [107.8;117.0]
*Diastolic blood pressure* (in mmHg)	56	81.2 [77.6;84.7]	78.6 [75.3;81.8]	55	80.4 [76.7;84.0]	78.8 [75.5;82.1]
*Triglycerides* (in mmol/L)	51	1.6 [1.2;2.0]	1.4 [1.2;1.7]	52	1.9 [1.4;2.3]	1.9 [1.5;2.3]
*HDL-C* (in mmol/L)	52	1.3 [1.1;1.4]	1.2 [1.2;1.3]	53	1.1 [1.0;1.3]	1.1 [1.0;1.2]
*HbA1c* (in %)	53	5.5 [5.3;5.7]	5.3 [5.2;5.4]	54	5.5 [5.3;5.6]	5.2 [5.1;5.4]

	T1 (men)			T2 (men)		
	*n*	Control (*n* = 21)	Intervention (*n* = 20)	*n*	Control (*n* = 21)	Intervention (*n* = 20)
*CRF* (in ml/kg/min)	38	37.5 [34.1;40.9]	39.1 [34.2;44.0]	38	38.2 [34.2;42.2]	40.8 [36.0;45.6]
*Waist circumference* (in cm)	41	85.1 [77.8;92.3]	84.8 [78.1;91.4]	41	89.1 [83.3;94.8]	85.4 [79.4;91.3]
*Systolic blood pressure* (in mmHg)	41	126.1 [120.2;131.9]	126.7 [123.3;130.2]	41	121.5 [115.7;127.3]	124.6 [120.5;128.6]
*Diastolic blood pressure* (in mmHg)	41	84.1 [80.5;87.8]	82.3 [78.7;85.8]	41	79.8 [75.7;83.9]	77.2 [74.3;80.1]
*Triglycerides* (in mmol/L)	38	1.7 [1.4;2.0]	1.8 [1.4;2.3]	41	2.5 [2.1;2.9]	2.2 [1.6;2.7]
*HDL-C* (in mmol/L)	38	1.1 [1.0;1.2]	.9 [.9;1.0]	41	1.0 [.9;1.1]	.9 [.8;1.0]
*HbA1c* (in %)	36	5.3 [5.2;5.4]	5.5 [5.3;5.8]	38	5.2 [5.1;5.3]	5.4 [5.2;5.6]

*Note*: Data are presented as mean [95 % CI].

*Abbreviations*: CRF, cardiorespiratory fitness; HbA1c, glycated hemoglobin; HDL-C, high-density lipoprotein cholesterol; T1, baseline; T2, post-intervention.

**Table 3:** Standardized direct and indirect effects on CRF and MetS components at T2.

	*R*^2^	Men	Age	Group (direct)	Group (indirect)	CRF T2	Modelfit		
							*χ*^2^/*df*	CFI	RMSEA
*CRF*	0.73	0.06 (0.379)	−0.21 (<0.001)	0.12 (0.022)			1.096	0.998	0.031
*Abdominal obesity*	0.66	−0.03 (0.720)	0.06 (0.160)	0.06 (0.362)	−0.03 (0.012)	−0.21 (0.008)	1.663	0.984	0.083
*High systolic blood pressure*	0.34	0.12 (0.177)	0.02 (0.840)	0.00 (0.972)	−0.01 (0.413)	−0.07 (0.549)	1.734	0.974	0.087
*High diastolic blood pressure*	0.38	−0.01 (0.983)	0.01 (0.871)	−0.07 (0.366)	−0.04 (0.011)	−0.29 (<0.001)	1.716	0.976	0.086
*Elevated Triglycerides*	0.36	0.23 (0.023)	0.13 (0.174)	0.09 (0.299)	−0.03 (0.025)	−0.23 (0.027)	1.215	0.992	0.047
*Lowered HDL-C*	0.40	−0.14 (0.120)	−0.04 (0.730)	−0.12 (0.192)	0.01 (0.300)	0.08 (0.437)	1.675	0.974	0.083
*Elevated HbA1c*	0.43	−0.23 (0.001)	0.24 (0.009)	−0.05 (0.539)	0.01 (0.117)	0.10 (0.180)	0.621	1.000	<0.001

*Note*: Effects are based on data with imputed missing values and presented as standardized path coefficients (*p*-value).

*Abbreviations*: CRF, cardiorespiratory fitness; HbA1c, glycated hemoglobin; HDL-C, high-density lipoprotein cholesterol; MetS, metabolic syndrome; T2, post-intervention.

## 4 Discussion

This study extends existing empirical evidence of the effects of exercise and sport on CRF and MetS (Pattyn et al., 2013; Batacan et al., 2017; Yang et al., 2009; Milanović et al., 2015; Lin et al., 2015; Ostman et al., 2017) to forcibly displaced individuals living in a refugee camp in Greece.

The intervention group improved their CRF significantly by 2.8 ml/kg/min more than the control group at T2. Older age was associated with lower CRF at T2. Thus, the age difference, with an approximately 4-years-younger intervention group, affected the outcome. Given that a 1 ml/kg/min increase in CRF corresponded to a 9 % decrease in the relative risk of all-cause mortality (Laukkanen et al., 2016), the observed improvements from the intervention were considered clinically relevant. This result aligned with the broad empirical evidence that regular participation in exercise and sport activities effectively enhanced CRF (Batacan et al., 2017; Milanović et al., 2015; Ostman et al., 2017). Previous studies showed that individuals with low fitness levels benefitted the most from enhanced CRF, whereby more than half of the reduction in all-cause mortality was observed when comparing the least-fit group to the second-least-fit group (Ross et al., 2016). Nearly two-thirds of the participants in our study possessed CRF levels below the 40th percentile at T1, with women showing notably lower CRF levels compared to men. Consequently, women may have derived greater benefits from the intervention than men. These findings could be attributed to the low physical activity levels reported among forcibly displaced individuals from Southwest Asia, often a consequence of migration-related socioenvironmental changes (Elshahat and Newbold, 2021). The observed low CRF levels, the enhancement in CRF through our intervention, and the reported benefits of improved CRF emphasize the potential of exercise and sport activities within refugee camp settings.

The prevalence of MetS in the total sample, though elevated, aligned with global estimates (Saklayen, 2018), implying that study participants were not disproportionately affected. This contrasted with some studies indicating a heightened risk of MetS among forcibly displaced individuals due to migration-related lifestyle shifts, such as changes in physical activity levels or dietary acculturation (Al-Rousan et al., 2022; Rosenthal et al., 2022; World Health Organization 2022). However, others cautioned against broad generalizations, emphasizing that the risk varies based on numerous sociodemographic and structural determinants. These included behavioral factors, origin, migration status, duration of residence, living conditions, migration policies, or accessibility to health services (Nisar et al., 2023; Agyemang and van den Born, 2022).

The intervention group in our study did not show stronger improvements in the MetS components than the control group. Although existing literature has demonstrated the effectiveness of exercise and sport on MetS components, the extent of this impact depended on the target group and nature of the intervention (Pattyn et al., 2013; Batacan et al., 2017; Yang et al., 2009; Lin et al., 2015; Ostman et al., 2017). As such, interventions of extended durations were more frequently associated with beneficial effects, implying that the length of the intervention was a critical factor in promoting changes in MetS components (Da Silva et al., 2020). The 10-week span of our study may thus have accounted for the lack of statistical differences between the intervention and control groups. Another explanation could be unconsidered risk factors. For instance, we noticed increased triglyceride levels in the male control group and decreased HDL-C levels in the female intervention group over the intervention period. Past studies have linked migration-related dietary changes, particularly adopting Western-type diets rich in processed foods, sugars, and saturated fatty acids, to a heightened prevalence of MetS (Pérez-Martínez et al., 2017). Our findings suggest the need for a more holistic approach beyond exercise and sport activities. Incorporating lifestyle changes like healthy nutrition could have a vital impact.

Although we did not identify a direct intervention effect on MetS components, CRF significantly facilitated the effect of the intervention on abdominal obesity, high diastolic blood pressure, and elevated triglycerides via an indirect pathway. This observation aligned with prior research, as CRF was consistently inversely associated with the incidence of MetS, cardiovascular diseases, and all-cause mortality (Ross et al., 2016). For instance, a study documented that individuals with high CRF faced a two-thirds reduced risk of developing MetS compared to those with low CRF (Laaksonen et al., 2002).

A dose-response relationship between increased physical activity, higher CRF, and reduced likelihood of MetS has been previously reported (Cheng et al., 2003; Sassen et al., 2009), which underscores the importance of promoting physical activity. In our intervention group, 73.5 % attended at least one session weekly, with 40.8 % meeting the bi-weekly recommendation. Thus, inconsistent adherence potentially accounted for the absence of noticeable direct effects on MetS components. Comparatively, exercise and sport sessions for forcibly displaced individuals reported low attendance (Elshahat and Newbold, 2021). Specifically, a prior study with forcibly displaced women noted a weekly participation rate four times less than ours (Sundquist et al., 2010). Considering that this intervention offered one session per week, compared to the five sessions provided in our study, this observation should be weighed against the frequency and utilization of the sessions. The results suggest that a wider range of exercise and sport activities may be necessary to achieve sufficient participation rates. The co-design approach used in our study may have contributed to these results. While our intervention reached a considerable number of individuals, the large standard deviation in participation rate pointed to heterogeneity among participants. Future studies should identify potential target groups within refugee camps who are less likely to participate regularly in exercise and sport activities and explore intervention strategies to enhance adherence among these groups. However, not all individuals may be drawn to exercise and sport but enjoy other forms of physical activity. For instance, walking was the most preferred physical activity among forcibly displaced Southwest Asians (Elshahat and Newbold, 2021).

This study had notable strengths. It responded to a pressing public health challenge by addressing the repeated call to meet the health needs of forcibly displaced individuals facing challenging living conditions (Médecins Sans Frontières, 2021). We successfully overcame several difficulties, including restricted camp access and numerous language barriers. While health promotion studies focused primarily on the mental health of forcibly displaced individuals (Purgato et al., 2021), our study was, to our knowledge, among the first efforts to examine the effects of a co-designed exercise and sport intervention on MetS components among individuals living in a refugee camp. By implementing a pragmatic design, we demonstrated the reach and effect of the intervention in a real-world context.

In contrast, the contextual setting led to a considerable number of dropouts and missing data, particularly in CRF, which may have influenced the main findings. Nonetheless, our results were consistent with a broad body of existing literature (Batacan et al., 2017; Milanović et al., 2015; Ostman et al., 2017; Ross et al., 2016; Laaksonen et al., 2002). Further limitations include the non-blinded design, which may have introduced bias affecting outcome assessments and data analysis. Given the study's pragmatic nature, systematic assessments of various control conditions throughout the intervention period, such as physical activity, individual intensity levels during sessions, or details on medication and dietary intake, were not feasible. The relatively short intervention length, paired with inconsistent adherence to the recommended bi-weekly participation, might explain the lack of observed effect on the MetS components. Overall, forcibly displaced individuals constitute a diverse population. Generalizing findings across this group requires caution (Nisar et al., 2023). Due to the sample size, we could not conduct subgroup analyses based on sociodemographic or health status criteria and determine whether specific groups benefitted more from the intervention than others.

## 5 Conclusion

The rising global prevalence of MetS similarly affects forcibly displaced individuals. Public health strategies must incorporate these individuals to prevent the emergence of health disparities. This study contributes to the long-standing call to address the health needs of individuals living under challenging conditions in refugee camps. Our study demonstrates that implementing exercise and sport activities in a refugee camp in Greece effectively improves CRF among forcibly displaced individuals, without directly affecting MetS components. However, enhancements in CRF are associated with decreased abdominal obesity, high diastolic blood pressure, and elevated triglyceride levels. While exercise and sport intervention provide one component to health strategies, they eventually need to be supplemented with broader lifestyle interventions and structures that enable an active lifestyle and promote a balanced diet.

## Funding

The authors acknowledge the financial support from the Swiss Network for International Studies (SNIS) and the universities involved in this study. The funding sources did not influence the design of the study, the collection, management, analysis, and interpretation of the data, the writing of the manuscript, or the selection of the journal.

## Declaration of generative AI and AI-assisted technologies in the writing process

During the preparation of this work the author(s) used ChatGPT-4 in order to improve language and readability. After using this tool/service, the author(s) reviewed and edited the content as needed and take full responsibility for the content of the publication.

## CRediT authorship contribution statement

**Florian Knappe:** Conceptualization, Formal analysis, Investigation, Methodology, Project administration, Supervision, Writing – original draft. **Konstantinia Filippou:** Conceptualization, Investigation, Methodology, Project administration, Supervision, Writing – review & editing. **Antonis Hatzigeorgiadis:** Conceptualization, Investigation, Methodology, Project administration, Resources, Supervision, Writing – review & editing. **Ioannis D. Morres:** Conceptualization, Investigation, Methodology, Project administration, Resources, Supervision, Writing – review & editing. **Emmanouil Tzormpatzakis:** Investigation, Writing – review & editing. **Elsa Havas:** Investigation, Writing – review & editing. **Harald Seelig:** Formal analysis, Methodology, Supervision, Writing – review & editing. **Sebastian Ludyga:** Methodology, Supervision, Writing – review & editing. **Flora Colledge:** . **Marianne Meier:** . **Yannis Theodorakis:** Conceptualization, Methodology, Writing – review & editing. **Roland von Känel:** Conceptualization, Methodology, Writing – review & editing. **Uwe Pühse:** Conceptualization, Methodology, Writing – review & editing. **Markus Gerber:** Conceptualization, Funding acquisition, Methodology, Project administration, Resources, Supervision, Writing – review & editing.

## Declaration of competing interest

The authors declare that they have no known competing financial interests or personal relationships that could have appeared to influence the work reported in this paper.
